# Streptococcal toxic shock syndrome in the intensive care unit

**DOI:** 10.1186/s13613-018-0438-y

**Published:** 2018-09-17

**Authors:** Marylin Schmitz, Xavier Roux, Benedikt Huttner, Jérôme Pugin

**Affiliations:** 1 Division of Intensive Care, Faculty of Medicine Geneva, University Hospitals of Geneva, University of Geneva, Rue Gabrielle-Perret-Gentil 4, 1211 Geneva 14, Switzerland; 2 Division of Infectious Diseases, Faculty of Medicine Geneva, University Hospitals of Geneva, University of Geneva, Rue Gabrielle-Perret-Gentil 4, 1211 Geneva 14, Switzerland

**Keywords:** Streptococcal toxic shock syndrome, Necrotizing fasciitis, Group A streptococcus, Sepsis, Bacterial toxins

## Abstract

The streptococcal toxic shock syndrome is a severe complication associated with invasive infections by group A streptococci. In spite of medical progresses in the care of patients with septic shock during the last decades, this condition has remained associated with a high mortality. Early recognition and multidisciplinary management are key to the care of patients with streptococcal toxic shock syndrome, with intensive and appropriate intensive support of failing organs, rapid diagnosis of infectious source(s), and surgical management. The epidemiology and risk factors for streptococcal toxic shock syndrome remain to be better studied, including the possible causal role of exposure to nonsteroidal anti-inflammatory drugs. In this review article, the authors review the current knowledge of streptococcal toxic shock syndrome and discuss the pathophysiology as well as its supportive and specific treatment.

## Clinical case

A 40-year-old woman known for type 1 diabetes presented to the emergency room with back pain, diffuse myalgia, asthenia, and fever (38.5 °C). She had been taking nonsteroidal anti-inflammatory medication for 5 days following a trauma of the left ankle. Physical examination showed hypotension with arterial blood pressure of 70/30 mmHg. The patient remained hypotensive despite fluid resuscitation with crystalloids and required continuous norepinephrine infusion. At the time of admission, the patient complained of important pain in the left limb, although this was associated with a normal clinical examination. A small skin defect on the left ankle in relationship with the initial trauma was, however, noted. The gynecologic examination was normal. Laboratory tests on admission showed elevated plasma C-reactive protein and procalcitonin levels (320 mg/L and 23 μg/L, respectively), an estimated glomerular filtration rate of 15 mL/min/1.73 m^2^, an absolute neutrophil count of 1.4 G/L with increased band forms, thrombocytopenia (63 G/L), increased thromboplastin time, and very elevated creatine kinase levels (143,000 U/L). The patient was admitted to the intensive care unit (ICU) with the diagnosis of septic shock, and empiric antibiotic therapy was initiated with piperacillin/tazobactam plus clindamycin. A CT scan of the limb did not show radiologic signs of necrotizing fasciitis, but the patient subsequently rapidly developed signs of arthritis of both ankles plus the right elbow, which required surgical lavage. Synovial fluid and blood cultures came back positive with group A streptococcus (*S. pyogenes*), and the antibiotic therapy was deescalated for high-dose IV penicillin G. In spite of the initial acute renal failure and rhadomyolysis, the patient did not need renal support therapy during the ICU stay, could be transferred to the ward after 6 days, and recovered without sequelae. The final diagnosis was a toxic shock syndrome due to group A streptococcus with septic oligoarthritis, most probably originating from a skin lesion and a left ankle trauma, in young woman with type 1 diabetes.

## Introduction

Streptococcal toxic shock syndrome (STSS) is a severe life-threatening condition complicating invasive infections by streptococci, mainly group A streptococcus (GAS, *S. pyogenes*) [1]. Streptococcal infections are frequent and can lead to a broad range of diseases from self-limited pharyngitis to severe diseases: bacteremia, pneumonia, meningitis, endocarditis, arthritis, sinusitis, and deep soft tissues infection, such as necrotizing fasciitis and myositis [2]. The STSS represents a severe complication of (mainly invasive) group A streptococcal infections, and less frequently due to other streptococcal species [3, 4].

Severe invasive GAS infections have been known for a long time, but GAS infections associated with shock and multiple organ failure have been first reported in the beginning of the 1990s after the description *princeps* of the staphylococcal toxic shock syndrome in 1978 [5–9]. In 1989, Steven et al. [1] reported a “toxic shock-like syndrome” among patients with scarlet fever associated with GAS. The term streptococcal toxic shock syndrome was further coined, in analogy to the staphylococcal toxic shock syndrome. A similar but distinct entity of STSS, caused by nongroup A streptococci, was also reported [10, 11].

The exact mechanism of STSS is not entirely understood but has to do with a combination of the effect of streptococcal toxins—enterotoxins with superantigen activity—other streptococcal enzymes and toxins, and the host response to streptococcal infection, a complex interplay between host immunity and pathogen virulence [12].

Admission to the ICU is usually necessary for patients with STSS. Treatment usually requires the control of the infectious source, which is particularly important in patients with myositis and/or necrotizing fasciitis, as well as the support of failing organs. There is controversy as to whether patients with STSS and multiple organ failure should be treated with polyclonal intravenous immunoglobulins (IVIG). A puzzling finding is that patients presenting with STSS have frequently taken NSAIDs, although the causal role of NSAIDs remains controversial. Herein, we review the current knowledge on the clinical presentation, and the treatment of STSS, which remains a severe, and frequently life-threatening condition in the ICU, sometimes requires debilitating surgical debridement.

## Epidemiology of streptococcal toxic shock syndrome

Group A steptococcus (*S. pyogenes*) is an aerobic gram-positive bacterium characterized by its beta-hemolytic activity (complete hemolysis in blood agar culture plates). Not all GAS strains carry genes and/or are able to release exotoxins with superantigen activity (see below). To establish the diagnosis of STSS, GAS need to be isolated from a sterile site [13]. Strains of GAS isolated from patients with invasive disease harbor predominantly types 1 and 3 M protein and secrete the pyrogenic exotoxin (superantigen) A, B or both. Nasopharyngeal mucosa and skin are the principal sites of asymptomatic GAS colonization. GAS are believed to enter into deeper tissues and the bloodstream by a rupture of an epithelial barrier, but has also the capacity to penetrate through intact membranes [12].

Streptococcal toxic shock syndrome has been reported among children and adults all around the world, but remains a relatively rare disease. Whereas sporadic cases are the rule, some clusters and outbreaks of STSS in closed environments, such as hospital and nursing homes, and even families, have been reported [14, 15]. Transmission of GAS causing STSS among family members has also been described [16, 17].

In prospective population-based surveillance data from Europe and Australia, the incidence of invasive GAS infections was around 3 cases/100,000 inhabitants/year [17]. Between 13 and 15% of patients presenting with invasive GAS infections develop STSS with a mortality rate ranging from 23 to 44% [16, 18]. Data from Center for Diseases Control (CDC) reported 309 cases of STSS infection, an incidence of 0.2 cases per 100,000 inhabitants/year, with a case fatality rate of 36% [18].

It is now well established that GAS infections, even when invasive, are associated with a low attributable mortality, unless they are invasive and meet the criteria for a toxic shock syndrome. Streptococcal toxic shock syndrome mortality is mainly influenced by the patient’s medical history, the site of infection, comorbidities, extremes of age, and the delay of diagnosis. The morbidity associated with STSS can also be very significant, particularly when extensive surgical debridement is needed in cases with necrotizing fasciitis, and as a consequence of organ failure in shocked patients, some of them might be permanent, such as respiratory and renal insufficiency [19, 20].

## Clinical presentation of streptococcal toxic shock syndrome

The CDC coined the definition of STSS in 2010 as “a severe illness associated with noninvasive or more frequently with invasive group A streptococcal infections” [13, 21]. Patients with STSS usually present with a series of symptoms due to a combination of toxin secretion and the primary focus of infection. Systemic symptoms of STSS are in great part related to the production and the secretion of GAS superantigen exotoxins, cross-linking the major histocompatibility class II and T cell receptors, present on antigen-presenting cells and T lymphocytes, respectively. Symptoms are therefore closely related to the response of stimulated immune cells secreting pro-inflammatory cytokines and vasodilating mediators, resembling to those observed in “classic” septic shock, due to endotoxins, for example. STSS may occur as a complication of GAS infections occurring at any site, but most frequently associated with soft tissue infections, such as cellulitis, myositis, and necrotizing fasciitis [22].

**Fig. 1:**
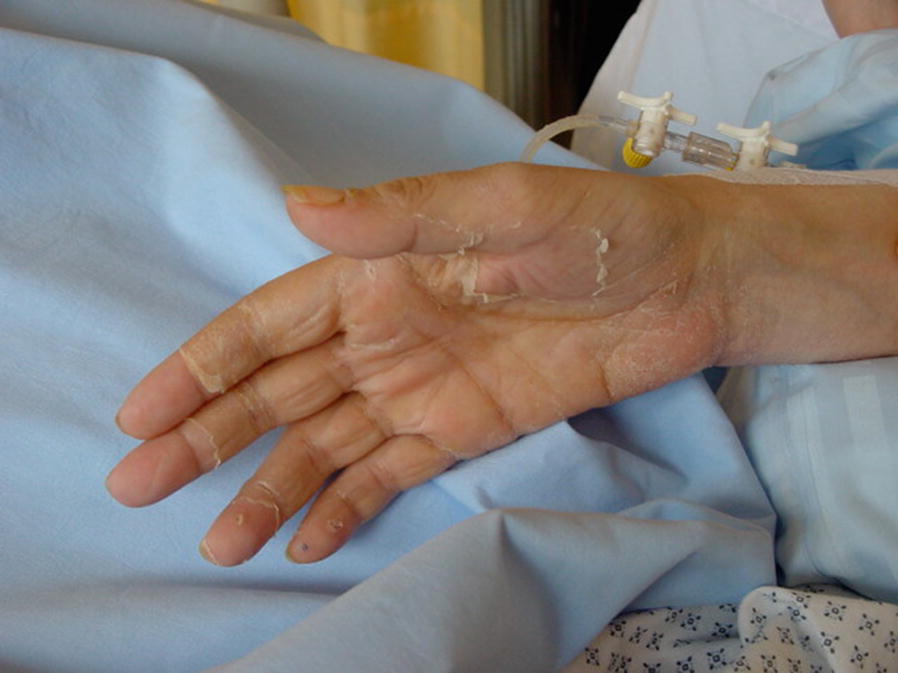
Palmar desquamation occurring a few days after STSS

Classically, three phases are described for the clinical presentation of STSS [2]. The first phase which precedes the onset of severe hypotension by 24–48 h. is a severe influenza-like illness characterized by high fever, myalgia, headaches, and chills. Nonspecific digestive symptoms such as nausea, vomiting, and diarrhea may also be present during this initial phase. Alteration of the central nervous system with delirium is reported in roughly half of patients. Skin lesions as possible streptococcal infection may be present. Examination of the skin and soft tissues is essential in the initial clinical evaluation of the patients, as well as during follow-up, up to four times per day, looking for tenderness, localized swelling, and erythema, or the more suggestive violaceous *bullae,* such as those observed in necrotizing fasciitis. An early and transient macular rash may be present, predominating on the upper chest [23]. Palmar and plantar desquamation is classical in STSS, but does not occur in all patients and is usually observed only few days after the in initial symptoms (Fig. 1) [23].

Soft tissues involved with GAS infections are usually very painful (hyperalgesia), frequently disproportionately compared to findings on clinical examination. Pain can be localized in a limb, but also on the abdomen, pelvis or thorax. The discrepancy between the intensity of the pain reported by the patient, and a normal (or quasinormal) clinical examination should alert the clinician for the possibility of a STSS. Changes in skin color and violaceous bullae are also highly suggestive of invasive *S. pyogenes* soft tissue infection. The clinical evidence for deep invasive infection will become more obvious as the illness progresses. Erroneous diagnoses, depending on the localization, are frequent. Classical initial misdiagnoses are deep vein thrombosis, limb ischemia, gastroenteritis, peritonitis, acute coronary syndrome, pericarditis, and meningitis.

In some cases, a skin lesion suggestive of GAS entry is visible early in the development of an invasive GAS infection and should be thoroughly searched for. This lesion can be as insignificant as a skin abrasion, often associated with localized redness, edema, but also hematoma or bullae. However, in more than half of severe GAS infections with STSS, such skin lesions will not be found [2].

**Fig. 2:**
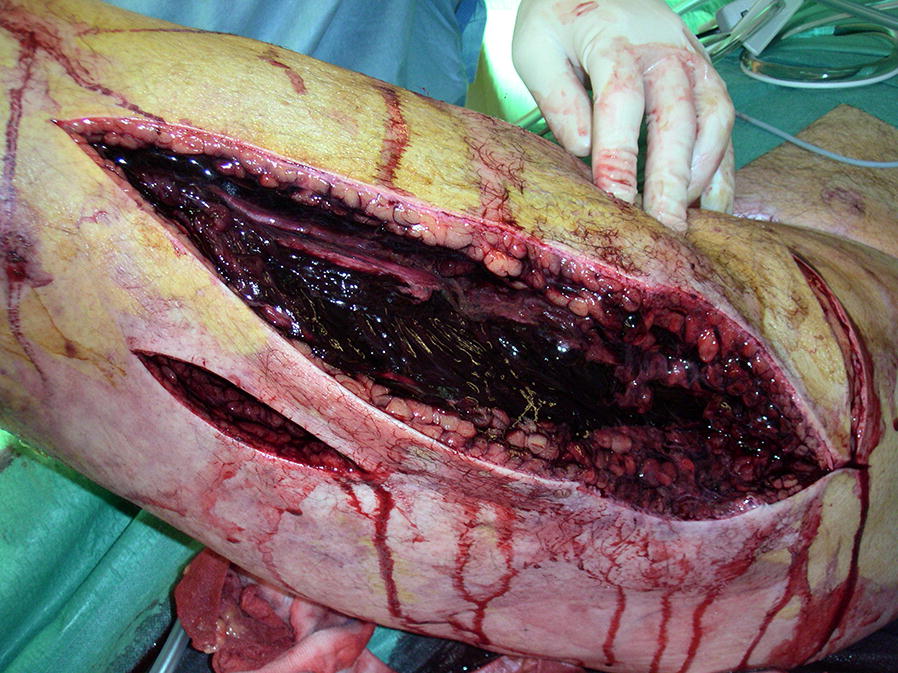
Muscle necrosis (black) of a thigh during surgical debridement in a patient with toxic streptococcal toxic shock syndrome due to *S. pyogenes* necrotizing fasciitis

The second phase of the STSS is characterized by systemic manifestations, such as tachycardia, tachypnea, and high fever. As said previously, pain is usually present in a limb, in the abdomen or the thorax, and is disproportionate compared to the clinical findings, even with deep invasive infection, such as necrotizing fasciitis (Fig. 2). Other possible and more frequent causes of infectious occurrences of fever should be ruled out, such as pneumonia, abdominal infection, or meningitis in case of neurological impairment. At this stage, computed tomography (CT) scan and/or magnetic resonance imaging (MRI) exams are usually useful to evaluate the soft tissue source of GAS infection, as well as to make a difference between skin infection and fasciitis. In case of doubt, surgical revision should be performed with biopsy of the fascia, and intensive lavage and debridement in case of infection and necrosis, respectively.

The third phase of clinical presentation is characterized by circulatory shock that can be sudden and profound, accompanied by multiple organ failure. Despite aggressive therapy many patients will die within 24–48 h of hospitalization.

The clinical criteria defined by the CDC are shown in Table 1 and are based on signs of shock, organ dysfunction, and skin involvement. Although used widely, many of the CDC criteria are not specific for STSS and are observed in sepsis or septic shock associated with other bacteria. Acute renal failure with elevated serum creatinine levels at the time of admission is, however, classical in STSS. In Table 2 are summarized symptoms, signs, and elements of clinical history suggestive of STSS reported in the literature.

**Table 1:** Clinical criteria for streptococcal toxic shock syndrome based on CDC definitions [13]

Clinical criteria: An illness with the following clinical manifestations^a^	
Hypotension defined by a systolic blood pressure less than or equal to 90 mmHg for adults or less than the fifth percentile by age for children aged less than 16 years	
Multiple organ involvement characterized by two or more of the following	
Renal impairment: Creatinin ≥ 2 mg/dL (≥ 177 µmol/L) for adults or ≥ twice the upper limit of normal for age. In patients with preexisting renal disease, > twofold elevation baseline creatinine levels	
Coagulopathy: Platelets ≤ 100,000/mm^3^ (≤ 100 × 10^6^/L) and/or disseminated intravascular coagulation, defined by prolonged clotting times, low fibrinogen level, and the presence of fibrin degradation products	
Liver involvement: Alanine aminotransferase, aspartate aminotransferase, or total bilirubin levels ≥ twice the upper limit of normal for the patient’s age. In patients with preexisting liver disease, a > twofold increase over baseline levels	
Acute respiratory distress syndrome: defined by acute onset of diffuse pulmonary infiltrates and hypoxemia in the absence of cardiac failure or by evidence of diffuse capillary leak manifested by acute onset of generalized edema, or pleural or peritoneal effusions with hypoalbuminemia	
A generalized erythematous macular rash that may desquamate	
Soft tissue necrosis, including necrotizing fasciitis or myositis, or gangrene	
Laboratory criteria for diagnosis	
Isolation of group A streptococcus	

^a^Clinical manifestations do not need to be detected within the first 48 h of hospitalization or illness, as specified in the 1996 case definition [24]. The specification of the 48-h time constraint was for the purpose of assessing whether the case was considered nosocomial, not whether it was a case or not

**Table 2:** Symptoms, signs, and elements of clinical history suggestive of STSS frequently reported among literature

Context of recent trauma, surgical intervention, skin lesion or NSAID intake	
Prodromal influenza-like symptoms	
Digestive symptoms (vomiting, diarrhea, abdominal pain)	
Severe pain, out of proportion is frequent, in the absence of evident portal of entry	
Signs of soft tissue infection (necrotizing fasciitis, gangrene, myositis, etc.)	
Generalized erythematous macular rash (early)	
Palmar and plantar desquamation (late)	
Multiple organ failure including two or more of the following: renal impairment, coagulopathy, liver involvement, acute respiratory distress syndrome	

The definition of “probable” and “confirmed cases” (CDC criteria) are the following [21]: A “probable case” is a case that meets the clinical case definition in the absence of another identified etiology for the illness, and with isolation of GAS from a non-sterile site. A “confirmed case” is a case that meets the clinical case definition and with isolation of group A streptococcus from a normally sterile site (e.g., blood, cerebrospinal, synovial, pleural or pericardial fluid).

## Laboratory investigations, microbiology, and imaging

The white blood cell count can be normal or only moderately elevated at the time of admission, usually accompanied by a marked elevation of circulating immature neutrophils (band forms). The diffuse capillary leak, together with IV fluid loading may contribute to low albumin levels. Hemolysins produced by GAS may cause a hemolytic anemia. A multiple organ dysfunction will reflect in organ-specific laboratory tests (increased creatinine in case of decreased renal function, low platelets and increased clotting times in case of coagulopathy, increased levels of transaminases for liver involvement, and hypoxemia in case of acute lung injury or ARDS) [2, 25, 26]. Increased creatinine levels at the time of admission are suggestive of STSS due to GAS, and more frequently observed than in other cases of “classical septic shock.”

A particular effort should be paid to seek for the presence of streptococci (GAS, essentially) from sterile since its presence defines “confirmed” cases of STSS (Table 1). The presence of GAS in non-sterile site defines “probable” cases in addition to clinical criteria. Similar to other types of infections, bacterial cultures (including blood cultures) should be performed rapidly, and prior to the administration of antibiotics. Depending on the suspected source of infection, the following sites should also be cultures: skin, deep soft tissue, peritoneal fluid, urine, sputum, pharyngeal swab, and synovial fluid. Antibiotics must be administered as soon as possible, just after the first blood culture, in case of high suspicion of streptococcal invasive infection with or without shock. Blood cultures will come back positive in about half of all cases with streptococcal STSS. Of note, staphylococcal toxic shock syndrome is rarely associated with positive blood cultures, being a “purer” toxin disease. Laboratory criterion for STSS diagnosis is “positive” if GAS is isolated in any microbiological test from sterile sites.

CT scans and MRI are helpful to identify the source of infection [27, 28]. MRI is more sensitive than CT for diagnosing skin and soft tissue infections but is less specific and therefore tends to increase the number of “false-positive” findings. The interpretation by the radiologist should always be taken with caution because radiological early signs might be benign: low local inflammation and no abscess formation, at least in the beginning, and absence of gas in the tissue, *S. pyogenes* does not produce gas [25]. A discordant radiological and surgical image is not infrequent at the early stage of fasciitis. The MRI is more specific for fasciitis. The presence of a thick (> 3 mm) hyperintense signal in the deep fascia on fat-suppressed T2 weighted or short tau inversion-recovery images are important markers for necrotizing fasciitis [27]. Although less predictive and informative, soft tissue echography can be useful in some cases since it can be performed at the bedside in the ICU unstable patient and can be frequently repeated [29]. An important message is that in case of a doubt, of a discordant radiological and clinical examination in a patient with systemic inflammation and/or shock, surgery should be considered without delay to evaluate the possibility of a necrotizing fasciitis, and surgical debridement with opening of the muscular fascias in cases of fasciitis. Surgical exploration is not only important for diagnosis (fasciitis remaining a diagnosis made by histopathology on a biopsy of a fascia [30], but is a cornerstone of fasciitis treatment [31].

Differential diagnosis of invasive GAS infections causing STSS include: gaseous gangrene, perineal Fournier’s disease, staphylococcal soft tissue infections and cellulitis, and synergistic cellulitis.

## Pathophysiology and predisposing factors

The pathophysiology of STSS is based on bacterial toxins. Superantigens are proteins that share the ability to trigger excessive and nonspecific T cell activation, therefore generating the massive secretion pro-inflammatory cytokines and other mediators producing capillary leak and arterial hypotension [32].

Staphylococci and streptococci are the two most common bacterial genera known to produce superantigens. Among streptococci, *Streptococcus pyogenes* (group A streptococcus), *S. dysgalactiae* (group C streptococcus), and *S. equis* (group G streptococcus) can produce exotoxins with superantigen activity [12, 32, 33]. The first streptococcal pyronegic toxin which appeared subsequently to be a superantigen was described in 1924 [34, 35]. Eleven different streptococcal superantigens (also known as streptococcal pyrogenic enterotoxins, SPEs) have been identified to date in *S. pyogenes*. Although they have different protein amino acid sequences and structures, they produce the same biological effects. They are single-chain proteins expressed as precursor molecules, which are then cleaved to release the functional extracellular toxin. They share sequence homologies in highly conserved regions called «family signature motifs» [12, 36]. The gene coding for superantigens can be present in streptococci, but may remain silent, with no expression of the toxin. The human factors triggering the expression of superantigens are largely unknown. Experimental studies have shown that GAS was able to modify gene expression depending on its environment, expressing virulence factors (such as superantigens) promoting the transition from superficial to invasive disease [37].

**Fig. 3:**
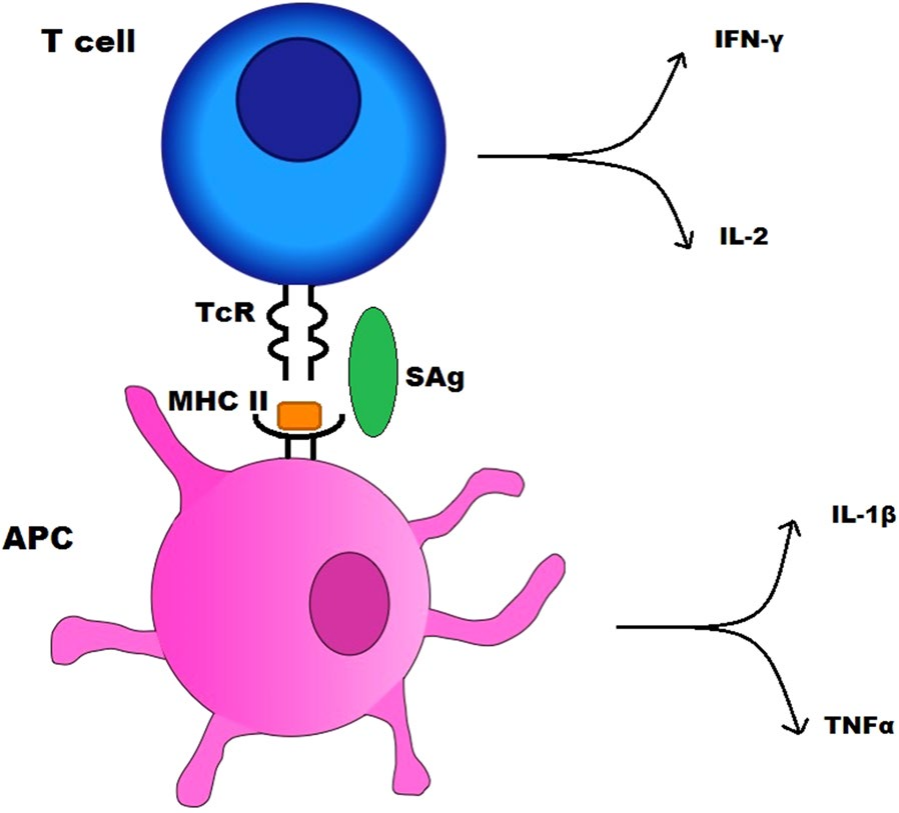
Schematic representation of T cell activation by a conventional peptide antigen (orange) and by a superantigen, binding unspecifically MHCII and T cell receptor, resulting to a massive, multiclonal release of T cell mediators and pro-inflammatory cytokines, in contrast to regulated, antigen-dependent inflammatory response during a conventional T cell activation, with activation of a single T cell clone. *APC* antigen-presenting cell, *TcR* T cell receptor, *MHCII* major histocompatibility class II molecule, *TNF-α* tumor necrosis factor alpha, *IFN-γ* interferon gamma, *IL* interleukin

The main characteristic of superantigens is their ability to bind to major histocompatibility (MHC) class II molecules outside of the antigen groove, and the Vß region of the T cell receptor, cross-linking those two receptors. This triggers the activation of both the antigen-presenting cell and the T lymphocyte, bypassing conventional mechanisms of MHC-limited antigen cell activation (Fig. 3). Since immune cell activation is not restricted to cells expressing and recognizing a single antigen, but rather polyclonal, cell activation is massive and explains the systemic pro-inflammatory activity, arterial hypotension, and end-organ dysfunction due to shock in patients with STSS [12, 38, 39]. The cross-linking of receptors by superantigens leads to the activation of up to 25% of lymphocytes as compared with < 0.1% for conventional antigen-triggered T-cell activation [40–42].

Aside from superantigens, *S. pyogenes* produces and secretes a wide variety of exotoxins and enzymes such as streptolysins, streptokinase, hyaluronidase, and DNAse such as streptodornase and chemokine proteases and toxic molecules that play undoubtedly an important pathogenic role in necrotizing fasciitis and STSS. In addition, *S. pyogenes* expresses surface virulence factors such as the M-protein (inhibition of opsonisation and phagocytosis), lipoteichoic acid (Toll-like receptor-dependent immune cell activation), protein F (adhesion to host cells) that are also considered as virulence factors [12].

Infants and elderly individuals carry the highest risk of invasive GAS infection. GAS is able to cause severe disease in otherwise healthy individuals, with between a fifth and a third of cases occurring in individuals with no predisposing risk factors to severe infection [19, 43].

Preexisting skin lesions are the most frequently identified risk factor for invasive GAS infection. Alcohol abuse, chronic lung disease, immunosuppression, intravenous drug use, heart disease, diabetes, cancer, varicella zoster virus infection, and recent child birth have also been identified as risk factors [44]. Interestingly, it has been demonstrated that MHC class II haplotypes influence the host susceptibility to develop STSS. The haplotype DR15/DQ6 is less commonly associated with STSS disease than the haplotype DR14/DQ5, for example [45–47].

Retrospective studies and case series describe the possible association between nonsteroidal anti-inflammatory drugs (NSAIDs) use and the development and/or the extension of serious invasive diseases due to GAS [48]. Where it is true that the association between NSAIDs consumption and STSS exists, it has been difficult until now to differentiate between a simple association (pain killer NSAIDs use in varicella, sore throat or because of pain in invasive GAS infection), and a causal effect or a participation of NSAIDs in the development of STSS. The association could also be that NSAIDs use in invasive GAS infection may mask initial signs and symptoms, and delay diagnosis and adapted treatment of invasive infections. Others have argued though that NSAIDs is independently associated with an increased risk for development of streptococcal toxic shock syndrome. Bryant et al. suggest that NSAIDs could delay muscle regeneration, induce cellular immunosuppression, and increase susceptibility to post-injury GAS infection. NSAIDs may accelerate GAS disease progression in established soft tissue infection and could reduce antibiotic efficacy [49, 50]. It has also been shown that endotoxin-treated volunteers had elevated tumor necrosis factor (TNF) levels, and decreased neutrophil functions when they had taken NSAIDs, and this may also be true in toxin-induced streptococcal shock [51, 52].

## Treatment

The management of STSS requires a multidisciplinary team involving intensivists, specialists in infectious diseases, microbiologists, and surgeons. Early identification of the disease, as well as rapid treatment, is key to minimize both morbidity and mortality in this deadly disease. Of extreme importance, a rapid source control and quick initiation of effective antibiotic therapy are both crucial. In the case of STSS, the admission to the ICU, and the initiation of supportive treatment of several dysfunctional organs, is usually necessary.

When a patient presents with elevated and persistent fever, pain in soft tissues out of proportion with the clinical examination, and/or signs of shock, the multidisciplinary team should meet at the bedside, including a surgeon, discuss the investigation strategy, usually CT, MRI and/or surgical review of the painful region (or the site with radiological abnormalities). The surgical approach has the advantage to look for fascia involvement visually, and to be able to perform a surgical biopsy and histopathology, deep bacteriological cultures, to open fascias if necessary, to check muscle viability underneath the fascia, and perform debridement of necrotic tissues. In case of necrotizing fasciitis and/or myositis, this aggressive surgical approach is the only one that may help stabilize the patient and save his life. The more unstable is the patient (heavy requirement of norepinephrine), the more rapid should this assessment be, and surgical debridement be performed.

In shocked patients, large volumes of crystalloids are usually required, together with significant doses of vasopressors. Invasive hemodynamic monitoring is generally useful in those patients to guide fluid loading, vasopressor treatment, and the possible requirement of inotropic drugs. Virtually all patients with STSS need intubation and ventilatory support, frequently develop ARDS, as well as renal failure requiring renal replacement therapy [2]. Acute kidney injury with elevated creatinine *at the time of admission* is frequently found in severe GAS infections and STSS, and when present should make the clinician think of this disease.

### Antibiotics

Surprisingly, *S. pyogenes* remains universally susceptible to penicillin despite the widespread use of penicillin for over 7 decades [53]. Some strains have, however, developed resistance to macrolides, tetracyclins and clindamycin. Penicillin G is bactericidal and remains, at high parenteral doses, the first-line treatment for infections due to SGA. In vivo, the efficacy of penicillin might be affected by the inoculum size. Clinical failures of penicillin alone have been reported. Due to the inoculum and steady-state volume of distribution disturbance, maximal parenteral doses of penicillin G are required (e.g., 4 mio IU/4 h.).

Clindamycin, a lincosamide antibiotic, is usually added to penicillin or aminopenicillin since it inhibits the protein synthesis by blocking the 50S sub-unit of the bacterial ribosome. It thus may therefore block the production of exotoxins such as the superantigens [54, 55]. The effect of decreased production of superantigens by clindamycin treatment has been demonstrated in animal studies [56]. Interestingly, penicillin and clindamycin both inhibit in vitro the production of the streptococcus superantigen pyrogenic exotoxins A (SPEA) and B (SPEB) isolated in *S. pyogenes* strains implicated in toxic shock syndrome, but the inhibition by clindamycin was significantly more important [57, 58]. Besides, the prolonged post-antibiotic activity and the lack of an inoculum effect (an increase in the minimal inhibitory concentration of an antibiotic when the number of organisms increases) may be desirable properties in the treatment of STSS.

The addition of clindamycin to penicillin may improve patient outcomes and reduce mortality [59]. Concentrations of the SPEA toxin were significantly lower 1 h after treatment when clindamycin or linezolid was added to penicillin, compared with penicillin alone. The mode of action of linezolid is similar to that of clindamycin, but its use in STSS is limited, and has no theoretical advantage over clindamycin [58]. The optimal duration of antibiotic treatment for STSS remains controversial and is usually guided by the clinical evolution, and by the need for recurrent surgical interventions. Duration of 2 weeks for the antibiotic therapy is often proposed, however, without any strong evidence supporting this duration of treatment. Again, it is important to stress that antibiotic therapy alone is not sufficient to treat and cure STSS. Antibiotic penetration into infected tissues, and sites of soft tissue and muscle necrosis is frequently very low, if not absent. This is essentially due to the poor vascularization of these sites, partly due to infection-induced microvascular thrombosis. Only surgical debridement of infected and necrotic tissues associated with high-dose systemic antibiotic therapy may improve mortality.

### Immunoglobulin

Lower levels of neutralizing antibodies against streptococcal toxins and the M-protein in patients’ plasma are correlated with invasive diseases of GAS [60, 61]. Case reports in the 1990s described a lower mortality in patients with STSS who benefited from polyclonal immunoglobulins [62–64]. These findings pointed at possible importance of antibodies in the protection against invasive diseases and suggested that addition of IV polyclonal immunoglobulins (IVIG) to the treatment may be useful as an adjunctive therapy. Possible mechanisms of action of immunoglobulins in STSS are: neutralization of toxins, improvement in bacterial opsonization, phagocytosis, and killing, as well as a possible immunomodulatory effect mediated by the interaction of Fc receptors and immune cells [65, 66].

The first report suggesting a lower mortality in the group of patients with STSS treated with IVIG was reported in an observational cohort study [67]. In a randomized, double-blind placebo-controlled study reported in 2003, patients with IVIG had a recovery of organ function significantly more rapid but no survival advantage [68]. This study was stopped prematurely because of a slow recruitment and was not powered to demonstrate the possible effect of IVIG on mortality. A recent randomized double-blinded placebo-controlled trial tested the effect of IVIG in necrotizing soft tissue infections (INSTINCT trial) [69]. There was no statistically significant difference between placebo and the interventional groups concerning the primary outcome (functional status assessed by the physical component summary (PCS) score of the 36-item short-form health survey (SF-36) 6 months after randomization) and the secondary outcome (mortality and multiple organ failure) [69]. However, patients suffering from documented STSS represented < 10% of the study population making it impossible to draw definite conclusions for this subgroup. Another recent retrospective study could not demonstrate a benefit of routine use of IVIG in necrotizing fasciitis [70]. Guidelines from the Infectious Disease Society of America (IDSA) state that additional studies testing the efficacy of IVIG in this indication are needed and also point to the fact that not all IVIG preparations are alike, in particular with the titers of neutralizing antibodies [71]. Therefore, the use of IVIG in STSS cannot be routinely recommended and should be discussed on a case-by-case basis. Plasmapheresis has been proposed, but level of evidence for its use is even smaller than that for IVIG, and only based on isolated cases reports [72].

## Prevention

### Chemoprophylaxis

Secondary invasive GAS disease has been documented to occur in individuals in close contact with the index patient. Carapetis et al. [59] report an incidence rate of developing an invasive GAS infection 2011 times higher than in the general population in Australia (95% confidence interval, 413–5929). The effectiveness of antibiotic prophylaxis on the risk of development of secondary invasive GAS infections remains, however, unclear [73–75]. The CDC recommends prophylaxis for contacts who have risk factors for the disease [76], and to advise contacts to rapidly search for medical help when presenting signs compatible with GAS infection.

### Vaccination

GAS is responsible for a high morbidity and mortality with diverse clinical manifestations. A safe vaccine which would not induce autoimmune pathology and cover different GAS strains would be of great interest. Despite various efforts, such a vaccine could not be developed to date [77–80].

### Preventing transmission

Transmission of GAS infection from patients with STSS to other patients or household contacts has been reported in healthcare settings. The CDC recommends contact and droplet plus standard precautions for the first 24 h of effective antimicrobial therapy in patients with severe GAS infection [81]. This recommendation is, however, not based on solid ground, and standard precautions are believed to be sufficient in many other institutions, including ours. Routine screening and prophylactic treatment of household contacts are currently not recommended [82, 83].

## Conclusion

The streptococcal toxic shock syndrome is an acute and severe systemic illness in part due to toxins with superantigen activity secreted by streptococci, mainly by group A streptococcus. The intensity, the rapidity of the development of shock and multiple organ failure on one hand, and cutaneous, fascia, and muscle necrosis, on the other hand, makes it essential to recognize and treat it rapidly. Clinical decisions need to be multidisciplinary, involving a team of intensivists, infectiologists, and surgeons. Surgical look and aggressive debridement of the infected site(s) is frequently required in necrotizing fasciitis. Antibiotic therapy should be given rapidly, associating high doses of parenteral beta-lactams plus clindamycin for its “anti-toxin” effect. Secondary surgery for lavage and debridement is also frequently needed to control STSS. Survival can be at the cost of impairment of limb function following muscle necrosis or amputations.

## Abbreviations

ICU: intensive care unit
STSS: streptococcal toxic shock syndrome
GAS: group A streptococcus
IVIG: polyclonal intravenous immunoglobulins
CT: computed tomography
MRI: magnetic resonance imaging
NSAIDs: nonsteroidal anti-inflammatory drugs
IDSA: Infectious Disease Society of America
TNF-α: tumor necrosis factor alpha
SPE: streptococcus pyrogenic enterotoxin
MHC: major histocompatibility
CDC: center for diseases control
APC: antigen-presenting cell
TcR: T cell receptor
IFN-γ: interferon gamma
IL: interleukin
